# Exploring Differences in the Content of Job Interviews between Youth with and without a Physical Disability

**DOI:** 10.1371/journal.pone.0122084

**Published:** 2015-03-23

**Authors:** Sally Lindsay, Anne-Marie DePape

**Affiliations:** 1 Bloorview Research Institute, Holland Bloorview Kids Rehabilitation Hospital, Toronto, Ontario, Canada; 2 Department of Occupational Science & Occupational Therapy, and Rehabilitation Sciences Institute, University of Toronto, Toronto, Ontario, Canada; University of Westminster, UNITED KINGDOM

## Abstract

**Objective:**

Although people with disabilities have great potential to provide advantages to work environments, many encounter barriers in finding employment, especially youth who are looking for their first job. A job interview is an essential component of obtaining employment. The objective of this study is to explore the content of the answers given in job interviews among youth with disabilities compared to typically developing youth.

**Methods:**

A purposive sample of 31 youth (16 with typical development and 15 with disability) completed a mock job interview as part of an employment readiness study. The interview questions focused on skills and experiences, areas for improvement, and actions taken during problem-based scenarios. Transcribed interviews were analyzed using a content analysis of themes that emerged from the interviews.

**Results:**

We found several similarities and differences between youth with disabilities and typically developing youth. Similarities included giving examples from school, emphasizing their “soft skills” (i.e., people and communication skills) and giving examples of relevant experience for the position. Both groups of youth gave similar examples for something they were proud of but fewer youth with disabilities provided examples. Differences in the content of job interview answers between the two groups included youth with disabilities: (1) disclosing their condition; (2) giving fewer examples related to customer service and teamwork skills; (3) experiencing greater challenges in providing feedback to team members and responding to scenario-based problem solving questions; and (4) drawing on examples from past work, volunteer and extra curricular activities.

**Conclusions:**

Clinicians and educators should help youth to understand what their marketable skills are and how to highlight them in an interview. Employers need to understand that the experiences of youth with disabilities may be different than typically developing youth. Our findings also help to inform employment readiness programs by highlighting the areas where youth with disabilities may need extra help as compared to typically developing youth.

## Introduction

There are 2.4 million working age Canadians with a disability, approximately 43,000 of whom are youth with disabilities [1], representing an untapped labor force in our current economy. A major obstacle to obtaining employment for individuals with disabilities can be at the job interview stage [2–5]. Employers often make quick judgments about applicants, including how they are dressed, their ability to answer questions, and general demeanor. People with disabilities may face additional challenges based on employer beliefs, which are often formed from a lack of experience with or understanding of people with disabilities. For example, some employers may be reluctant to hire individuals with disabilities for fear of costly workplace accommodations [6]. Individuals with disabilities also need to decide whether and how to disclose their condition because employers are not permitted to inquire about this information in a job interview [7].

Employment readiness programs for people with disabilities have taken different approaches. For instance, Verhoef et al. [8] took a multidisciplinary approach with their employment training focusing on work, self-care and leisure. These researchers found increased participation in paid employment and more community integration after training as shown by changes in living arrangements and leisure activities for those with disabilities [8]. Other researchers have focused specifically on interview training and found that interviewees responded positively to this method as indicated by their quantitative and qualitative responses [9]. In another study, employment readiness increased quality of life and attitudes about work among youth with disabilities [10]. Targeting youth with disabilities is important because employment experience in high school is often a predictor of employment in adulthood [11]. Many youth with disabilities do not receive the training and the preparation in high school needed to maintain employment in adulthood [12–13]. Thus, there is a need to educate youth with disabilities about the employment process, particularly how to market their skills and experiences in an interview [4, 13].

This study addresses an important gap in the literature by examining the content of job interviews of youth with disabilities compared to those with typical development. First, little is known about job interview performance, and specifically the content of answers within a job interview among youth with disabilities compared to their typically developing peers. Most research focuses on adults while less attention has been paid to youth. Although research consistently highlights that youth with disabilities have poorer employment outcomes compared to youth without disabilities [4–5], we lack insight into the specific areas they may need further help and support in obtaining competitive employment. One key hurdle is the job interview and thus, the focus of our paper.

Improving employment of youth with disabilities is critical given the employment participation rate for adults 20 to 24 years old is 63.7% for those with disabilities and 81.5% for those with typical development [14]. Employment rates are even lower for youth 15 to 19 years old: 40.1% for those with disabilities compared to 51.4% for those with typical development [14]. In the current study, we developed a mock job interview based on input from representative employment sectors that regularly hire youth [4]. The objective of this study was to explore the similarities and differences among youth with disabilities on a mock job interview compared to their typically developing peers. Understanding the content of job interviews may highlight any areas where individuals with disabilities may perform differently than those with typical development and in turn, how these areas may present barriers to employment. Our research will inform employment readiness programs and educate employers about the potential challenges associated with this recruitment method for those with disabilities.

## Methodology

### Design

This paper is part of a larger, multi-method (qualitative and qualitative), cross-sectional observational study on employment readiness among youth with a disability [5, 13] where we interviewed employers and employment counselors (n = 19), and youth (n = 31). For this paper, we used a content analysis of the qualitative responses of mock job interviews with 31 youth (16 typically developing and 15 with a physical disability). Mock job interviews were developed after interviewing a sample of job counselors and employers from industries where youth typically work (e.g., retail, accommodation and food services, education services, arts, entertainment and recreation) [15], which helped us to gain an understanding of the desirable skills that employers look for when hiring youth (see reference 5 for further details on the development of job interview content). We used this information to design our mock job interviews, which were pilot tested with four youth (two with typical development and two with a physical disability) [5]. Youth also completed a brief demographic questionnaire that asked about age, gender, work experience, type of disability, means of transport, job interview, volunteer experience and current employment status [5].

### Data collection and participants

Ethical approval was obtained through the University of Toronto and Bloorview Research Institute Research Ethics Boards. Youth were recruited through advertisements at a pediatric rehabilitation hospital and community centers. Information packages were also sent from the rehabilitation hospital to youth who were thought to meet the inclusion criteria. Youth had ample opportunity to decide whether they wanted to take part in the study (i.e., more than one week). If they were interested and consented to take part, they arranged a time to participate in the mock job interview with the researcher. All participants completed a written consent form prior to taking part. Informed consent from next of kin on behalf of the youth enrolled in our study was not required from our research ethics board. However, the research team assessed capacity to participate through a discussion with the youth (i.e., asking why they wanted to participate; do they understand what the study is about; and what is good or potentially difficult about being in the study). The ethics committees approved this consent procedure.

The content and delivery of our mock job interviews was evidence-based [4, 5, 13]. A professional actor who has experience in working with disabled youth played the part of the employer and led the interview. All participants were aware that this was a mock interview and not a real job interview. Each participant was asked the same questions in the same order [5]: (1) “Tell me a bit about yourself” (2) “What skills or experience do you have for this position” (3) “What do you consider to be your biggest strength” (4) “What do you consider to be an area for improvement” (5) “Tell me about a time you did something that you are proud of” (6) “Tell me about a time when you worked with someone who was not doing their share of the work” and (7) “How do you cope with this situation”. The situation involves the following: “You are a Greeter and Customer Service Specialist at our store. You just noticed a teenager spill his can of coke at the entrance of the store and walk away. There are three customers entering the store who need to be greeted and you reach into your pocket and realize you are out of happy face stickers to seal their bags. At the same time you receive a page that your manager who wants to speak to you. Tell me what you would do and please justify your decision-making process” [5].

Inclusion criteria of the youth involved: has a diagnosed physical disability without a cognitive impairment; currently enrolled in grade 11 or 12 of an academic/applied university stream within high school; and currently attending an integrated high school [5]. Our comparison sample also met the above criteria but they did not have a disability. None of the participants required accommodations to complete the data collection. However, it is important to note that the set-up of the interview room was accessible for people using a mobility device.

Data were collected on several different dates, depending on the availability of the actor and participants, from December 2012 to November 2013. The mock job interviews were held either in a meeting room within the rehabilitation hospital or within a community center. The same set-up for each mock job interview was created regardless of the setting. Only the actor and a researcher (who videotaped the interview) were present in the room at the time of data collection. Interviews lasted on average 6.85 minutes for typically developing youth and 8.38 minutes for youth with disabilities [5].

### Data analysis

All mock job interviews were audio recorded, transcribed verbatim and entered into NVIVO, version 10. Our content analysis drew on principles according to Elo and Kyngas [16]. Content analysis is a descriptive approach in coding and interpretation of the quantitative counts of the codes [16]. It involves systematic coding and categorizing to understand trends and patterns, their frequency and relationships [16–17]. This approach is suitable for simple reporting of common issues that are mentioned in the data where the purpose is to document the content of what is said and by whom [18].

The first stage of the content analysis involved the researchers immersing in the data to obtain a sense of the whole (by reading through each transcript several times), selecting the unit of analysis (i.e., individual participant responses to each question), deciding on the type of analysis (i.e., manifest content) and developing the categories [17–18]. The second stage involved open coding where two of the investigators and a research assistant independently read and coded each transcript while noting themes and patterns for each question. Each reviewer compared and contrasted their list of categories until consensus was reached. We created categories and grouped the codes under higher order headings and then developed a general description of the themes through generating categories and sub-categories while abstracting representative quotes [17–18]. Then the categories were organized by question and type of participant (youth with or without a disability) (see Table 1). An additional two members of the research team read through a sample of the transcripts to verify the categories and coding framework. Once the coding framework was finalized a research assistant applied the codes to the transcripts using line-by-line coding [17].

**Table 1:** Number of participants indicating a theme by question.

Question	Themes	Typical	Disability
**1. About yourself**	School	10	10
	People skills	5	6
	World experience	1	0
	Family	3	0
	Job experience	8	1
	Volunteer experience	6	3
	Problem solving	1	0
	Organizational skills	2	1
	Hobbies	2	0
	Sports/teams	4	0
	Career path	1	0
	Responsible	1	0
	Disability	0	5
	Confident	0	1
**2. Experience for the position**	Job/volunteer	9	5
	Customer service	10	7
	Communication	6	9
	Taking directions	1	0
	Organizational	2	1
	Punctual/reliable	2	0
	Working independently	1	0
	Hard working	1	0
	Problem solving	1	0
	Friendly	0	1
	Disability	0	1
**3. Area of strength**	People/communication	9	9
	Determination/outgoing	1	1
	Customer service	3	0
	Teamwork	3	0
	Independent	1	0
	Comprehension	1	0
	Disability	0	1
	Adaptable	0	1
	Math	0	1
	Organized	1	0
	Multi-lingual	0	1
**4. Area for improvement**	Trouble focusing/attention	2	1
	Patience	2	0
	Punctuality	2	2
	Negative statement	1	0
	More work experience	1	0
	Time management	3	1
	Knowledge of store	1	0
	Communication	1	0
	Being nervous	0	1
	Organizational skills	0	1
	Directional skills	0	1
	Initiating conversation	0	2
	Self-confidence	0	1
	Inappropriate example	1	1
	Nothing	1	1
	Not sure	0	1
**5. Something proud of**	Problem-solving	3	1
	Helping someone	6	2
	School achievement	3	5
	Organized/disciplined	1	0
	Taking initiative	1	1
	Independent	1	0
	Sport achievement	4	1
	Organized an activity	1	1
	Fixed computer	0	1
	Religious event	0	1
**6. Giving feedback**	Direct	7	6
	Indirect	3	0
	Negative	3	1
	Inappropriate/irrelevant	3	7
**7. Solving problem-based scenario**	Provided rationale	9	3
	Cleaned spill first	8	7
	Greeted customers first	6	4
	Paged manager first	2	4

Note: Typical = youth with typical development; Disability = youth with disability

The final stage of data analysis involved reporting the results of the previous stages. Quotes that were representative of each theme, by question, were abstracted. The research team discussed and approved the selection of quotes to ensure representativeness. Table 1 illustrates the range of categories arising out of the interviews and the number of times each participant mentioned a theme for each question. The purpose of this was to develop a better understanding of whether youth with and without disabilities differed in their answers and if so, within which questions.

### Sample

The sample consisted of 31 youth: 15 with a physical disability (9 males, 6 females) and 16 typically developing (6 males, 10 females). Ten of the youth had cerebral palsy, and the rest had various diagnoses including muscular dystrophy, myotubular myopathy, central core myopathy, scoliosis, spinal cord injury and Gullian-Barre syndrome. Eleven of the youth with disabilities used a wheelchair or walker. The mean age for youth with disabilities was 17.06 years while for typically developing youth it was 16.75 years. There were no significant differences in socio-demographic characteristics (e.g., gender, age, level of education) [5]. In regard to ethno-cultural status, our sample was reflective of the culturally diverse city where this data was collected. Fourteen of the youth were from an ethnic minority background (eight with a disability and six without).

## Results

We found several similarities and differences between youth with disabilities and typically developing youth. Similarities included giving examples from school and emphasizing their “soft skills” (i.e., people and communication skills) and giving examples of relevant experience for the position. Both groups of youth gave similar examples for something they were proud of but fewer youth with disabilities provided examples. Differences in the content of job interview answers between the two groups included youth with disabilities: (1) disclosing their condition; (2) giving fewer examples related to customer service and teamwork skills; (3) experiencing greater challenges in providing feedback to team members and responding to scenario-based problem solving questions; and (4) drawing on examples from past work, volunteer and extra curricular activities. The content of the findings are presented in order of the questions asked during the mock job interviews. Table 1 highlights the number of participants that mentioned a theme within each interview question.

### Introductions and “About yourself”

The first question asked youth to tell a bit about themselves. Youth with disabilities and those without both mentioned school and many spoke about their soft skills including people and communication skills. Specifically, 10 typically developing youth and 10 youth with a disability gave examples drawing on their school experiences within their description of themselves (see Table 1). For example, one typically developing youth explained, “My name is [name]. I’m 16 years old. I’m currently in grade 11. I go to [school name]. I enjoy reading and helping people” (#10). Similarly, one youth with a disability stated, “I’m 18 years old. I’m in high school and I just love being around people” (#6). Compared to youth with disabilities, those with typical development often mentioned their previous work (eight typically developing youth versus one youth with a disability) or volunteer experience (six typically developing youth versus three youth with a disability) within this introductory question. For example, one typically developing youth explained, “I go to [school name] and I’m currently working as a camp counselor for the [company name]” (#18). Six typically developing youth also described problem solving and other skills learned through extra curricular activities (e.g., sports, hobbies) and how this position might fit within their career path (see Table 1). One youth with typical development said, “I like working in retail. Also I like all sorts of sports ranging from basketball to hockey” (#22).

Youth with disabilities often did not have as much work and volunteer experience to draw upon. Instead, many youth with disabilities spoke about their how their condition affected them. For example, “I’m 18 years old and I was in the hospital for about a year and I’m trying to get back to normal” (#12). Another youth with a disability stated, “I’m seventeen years old. I have cerebral palsy and I’m a grade twelve student. I can’t think of anything” (#32). Five youth tried to spin their disability into a positive by showing how the job would increase their independence:

I have a physical disability, which is muscle weakness…I haven’t done any work lately but I am looking for some, just to be able to take care of myself for the future and I think this job would be really helpful and you know something I like to do (#29).

Although the introductory question did not ask for this directly, some typically developing youth provided the interviewer with their reason for applying for the current position. Reasons provided by typically developing youth included saving money for college tuition, obtaining summer job experience and perceived match between the job and their personality.

### Experience for the position

The second interview question asked about what relevant experience youth had for this particular position. More youth with typical development spoke about their job and volunteer experience than youth with disabilities (nine versus five, see Table 1). For example, one typically developing youth stated, “I used to work at [company], and it was similar kind of job description. I would greet customers and then I’d help them with their transaction, like accessing the machine for credit or debit and handling cash” (#18). Somewhat surprising, slightly more of the youth with disabilities (n = 9) talked about their people and communication skills compared to typically developing youth (n = 6). For instance, “For this position, I’m good with people and for greetings; I can show people around; I have a good memory” (#29). Another youth with a disability stated:

Probably keeping the somewhat cheery attitude. For that it seems to be if you can know how to smile, know how to show that this is a good place, I’m happy to work here; other people would say that this is a good place we can shop (#11).

Likewise, one typically developing youth described their ability to take direction, problem solve, be punctual and reliable. For instance, “I can fulfill my tasks, making sure it’s on time. I’m punctual and also able to adapt to any environment” (#17). Some youth with disabilities struggled to provide specific examples of their skills and experiences. For example, “I’ve done a lot of volunteer service, so I have experience working with people of all ages; that’s kind of about it” (#34). Interestingly, one youth with a disability described how having a disability would be an asset for the position: “This job requires a lot of communication skills, so being in a wheelchair I have to communicate very well with others to make sure that my needs are met” (#8). Some other examples that typically developing youth gave for relevant experience for the position included organizational skills (two participants), being reliable and punctual (two youth), taking directions (one youth), and able to problem-solve (one youth). Youth with disabilities did not mention these skills for this interview question.

### Greatest strength

When youth were asked about their greatest strength both groups often gave examples of people and communication skills (nine participants with a disability and nine typically developing youth). One typically developing youth said, “My biggest strength would be my co-operation skills. I am able to work with people really easily and an example would be, last year I participated in the [provincial] skills competition” (#24). Some typically developing youth linked their strengths to aspects related to the current job such as customer service (three participants) and teamwork (three participants). For instance, “I can convince someone to buy something that people probably wouldn’t buy” (#20). Another typically developing youth stated, “Being independent because my employer doesn’t really have to look at me as much and I can just do whatever by myself and not cause that much problems” (#23). Typically developing youth also talked about following instructions from supervisors (one participant), being reliable (two participants) and organized (two participants). Similarly, nine youth with disabilities described their people skills as strengths: “I guess a strength, would be talking to people. I have given speeches in front of groups of people before” (#32). Another youth with a disability explained:

I’m a people person and I’m most willing to be very assisting in all my capability—as hard as I can and as much as I can to help out a customer or person in need and I think that would be very helpful in working here as an employee for such a customer based environment (#31).

One youth with a disability connected his strength to his disability: “As a person with a disability you often have to ask for help from others so you learn to be very outgoing and adaptable towards other people” (#6).

Occasionally, youth with disabilities provided examples of strengths that were irrelevant to the job or not obvious in terms of the transferable skills they provided. Some examples included: “Okay strength,—I sing” (#29) and “Math, I get good grades in school” (#9).

### Area for improvement

A fourth interview question asked youth to describe an area for improvement, which brought a wide range of answers. Both groups of youth mentioned punctuality (two youth with and without a disability) and time management (three typically developing youth and one youth with a disability) were areas for improvement. For instance, one typically developing youth stated, “I’m a bit late sometimes, like going to school I might be late. So I might have to improve on that” (#13). Both groups of youth also mentioned they would like to improve time management and organizational skills. One typically developing youth explained, “I believe I am very efficient but then there’s always room for improvement and if I were to improve it—it would be of great benefit to not only me but your company” (#25). Other responses to the question included increasing knowledge about the company and products, communication skills and more work experience. For some youth with disabilities, the area they identified as needing improvement was more personal than job-specific. For example, “I tend to get a bit nervous. I would have to present myself in a calm, professional manner” (#8). Another youth reported, “I don’t have a lot of confidence in a lot of things” (#30). Two youth with disabilities described problems with initiation in meeting a new person. For example, “Probably my initiation because to kind of persuade the customers, I need to initiate talking to them and if I don’t have initiation then I’m not going to get any salary and I’m not going to get any customers” (#12). Other youth with disabilities mentioned areas for improvement without a clear explanation of how or why it should be undertaken.

Overall, our results suggest that youth with disabilities often gave a more personal example while typically developing youth focused on more job-specific tasks. Some typically developing youth also turned their area for improvement into a positive twist or offered a solution of how they are working toward enhancing this skill. For example, one typically developing youth explained, “I think I try to take on a lot of tasks so I guess I could delegate my time a bit better” (#18).

### Something proud of

A fifth interview question asked youth to describe something they were proud of. We found that slightly more youth with typical development than those with disabilities mentioned problem-solving skills (three typically developing youth versus one youth with a disability) and situations where they helped someone (six typically developing youth versus two with a disability). For example:

I had a volunteer position as a volunteer coordinator at a company and I was doing an event and it was a registration and it was the most stressful thing I’ve ever experienced. There were 12 people asking me what to do, and they were all older than me—I was 15 years old at the time, and I was coordinating about ten 30-year-old volunteers and I was in such deep water, and I don’t even know how it happened, but by the end, everything had been organized, and I’d done my job perfectly (#4).

Four typically developing youth also described their extracurricular activities and sports (compared to one youth with a disability). A typically developing youth explained:

I joined the Air Cadets. So it’s a military based youth program and I was proud because it took a lot of strength for me to go because they’re really strict and you have to be really managed. You have to prepare your uniform, you need to be there on time; you need to participate in different activities (#16).

Meanwhile, five youth with a disability versus three without described events they had organized or specific achievements at school. For example, “I got a scholarship that I was very proud of and then I was presented with an award” (#8). Another youth with a disability said, “I spoke in front of the whole school we elected for group council” (#32). For other youth with disabilities they talked about situations where they advocated for themselves:

There was this place that I had to be, it was a volunteer position actually it was downtown and that day I had booked Wheel-Trans [public wheelchair transportation bus] the night before and I had it all set up and everything but actually the day of, it never showed up. So I actually took the initiative myself to call the company, ask them what was going on. So, I was really proud of, that I could problem solve and take action in my own hands and actually take care of everything and still be on time so (#27).

Overall, both groups of youth gave similar responses in terms of something they were proud of but youth with disabilities tended to have fewer examples.

### Giving feedback

A sixth job interview question asked youth to describe a situation where they had to provide feedback to someone who was not doing their share of the work. Most youth (seven typically developing and six with a disability) described how they would provide direct feedback in a positive manner to group members who were not doing their share of the work. A youth with typical development explained:

Once I was on the debate team and there was a girl, who hadn’t done any of the work, and I’d done all of it, and I just had to—I just asked her nicely if she would do more. Not, not in a ‘you haven’t done anything’ way, more in a ‘would you mind helping’ way (#4).

Typically developing youth also emphasized the importance of telling team members how to improve their performance instead of criticizing their behavior. Similarly, youth with disabilities emphasized the importance of talking to team members about the issue. Some provided examples based on their work experience. Other youth with disabilities provided examples from school projects:

I was once in a group project at school, my friend wasn’t putting in the work so I had to have a talk with him and tell him that they need to put a little more work in the project so we could both get a good mark (#34).

Other youth with disabilities did not provide specific information about how they would confront their team members. For example:

Yeah, well I because we have some students at school who don’t always do their work on time I’m one of those people who does often have to tell people to get their work done before they can move on to anything else (#32).

Seven youth with disabilities gave responses that were irrelevant to the job: “My brother, I usually give him feedback on homework. My brother co-operates with me so it’s alright” (#9).

In sum, although there were similarities between both groups of youth regarding providing examples of direct positive feedback, more of the youth with disabilities gave an inappropriate or irrelevant answer.

### Scenario Problem-solving

This problem-solving scenario-based question involved three tasks with participants having to describe the order in which they would complete each of the following tasks and their reasoning for doing so: greeting customers, responding to their manager’s page and cleaning a spill in front of the store. Although there was no right or wrong answer, more of the typically developing youth said they would clean the spill as their first step in the problem-solving scenario while youth with disabilities described they would greet customers. Differences were also found in youth with disabilities who often did not provide a rationale for the steps they took in the problem-solving scenario (three youth with disabilities versus nine typically developing youth). For example, a youth with a disability explained, “I would choose to greet the customers first—hand them the flyers, greet them; pick up the can and throw it out, then I would page back my supervisor and ask them what they would like to talk about” (#11). Youth with disabilities often did not ask for help or delegate responsibilities to co-workers during this problem-solving scenario.

## Discussion

Our findings show that youth with disabilities were similar in many ways to their typically developing peers regarding the content of their answers within a mock job interview exercise. This is encouraging news and helps to dispel many of the myths and misconceptions about employment and people with disabilities. Similarities between the two groups of youth included drawing on examples from school and emphasizing their “soft skills” (i.e., people and communication skills) and giving examples of relevant experience for the position. This finding complements other research showing that soft skills, including being able to work in a team and communicating effectively are desirable among youth employers and are increasingly required in some of the fastest growing job sectors [13]. Soft skills are also particularly important for youth who often lack hard skills obtained through work experience [13, 19]. A second area that both groups of youth gave similar examples included a question asking youth to tell about something they were proud of; however, youth with disabilities gave fewer examples. This could be a result of youth with disabilities having fewer employment and extra curricular experiences to draw upon [5,19].

There were four notable differences within the content of the job interview exercise between youth with disabilities and typically developing youth. The first key difference involved youth disclosing their condition. Compared to typically developing youth, those with disabilities provided personal information, including about their condition, when asked to tell about themselves. Disclosing a condition within an interview setting is a complex decision because it is critical to provide employers with an understanding of their capabilities and possible accommodation needs, however, it could also result in discrimination or stigma [4]. Determining whether to disclose depends on the nature of the disability, such as whether someone has a visible or invisible condition [19, 20]. Within a job interview not all individuals with a disability are perceived in the same way. For example, one study shows that among individuals with physical disabilities, those who required the use of crutches were rated more qualified for the job than those who required the use of a wheelchair [3]. Thus, educating employers about disability is critical, particularly in cases where employers have no previous experience with hiring people with disabilities. Some research shows that individuals with disabilities are rated more favorably when disclosure about their condition occurs early instead of late in an interview [20]; however, research in this area has mixed results [4] and more research is needed on the disclosure process.

The remaining three differences between youth with and without a disability regarding the content of their answers in a job interview were probably a result of lacking job and volunteer experience to draw upon. Youth with disabilities commonly encounter barriers in finding employment and volunteer experience, which could contribute to their differing or lack of relevant answers in a job interview [5, 13]. Specific differences regarding their answers compared to typically developing youth included: (1) giving fewer examples related to customer and teamwork skills; (2) greater challenges in providing feedback to team members and responding to scenario-based problem solving questions; and (3) fewer examples from past work, volunteer and extra curricular activities.

Our findings are similar to Lindsay et al [4], who report that youth with disabilities had less work experience than youth with typical development. The consequence of lacking experience within an interview context was that youth with disabilities were less able to speak about their qualifications (i.e. related experience) and how they were suited for the position. Youth with disabilities also had difficulty providing a rationale for the steps they would take in our problem-solving scenario question. It is critical for youth with disabilities to seek out opportunities in their communities to obtain more work and volunteer experience. In Ontario, Canada, where this study was conducted, the Ontario Ministry of Education [21] requires that all students complete 40 hours of volunteer work to receive their secondary school diploma. Thus, youth with disabilities are encouraged to seek relevant volunteer placements so they can speak about these experiences in future job interviews.

The lack of work experience found among youth with disabilities may be compensated to some degree by promoting how their condition may provide beneficial experiences and add to a diverse work environment. That is, employers often report that workers with disabilities are honest, punctual, accept feedback, have low absenteeism rates and strong work ethics compared to workers with typical development [22]. Research also shows that consumers often look favorably upon companies that hire individuals with disabilities because it portrays an image of caring about their workers [23]. We found in our sample that only a few youth spoke about how their disability improved their ability to communicate and adapt to different environments. If youth feel comfortable in disclosing their disability, they should be encouraged to provide such information so employers can better appreciate the benefits they bring to the workplace. However, youth should also be aware that disclosure could be a double-edged sword if employers have negative attitudes towards people with disabilities. There is a need for job training programs to help youth gauge when, how and how much to disclose about their disability in an interview.

Knowing your ‘transferable’ or marketable skills and being able to connect your skills and previous experience to the current position are essential because employers expect workers to have certain skills in place prior to employment and want employees to be able to ‘sell’ these skills during an interview [4, 19]. We found that typically developing youth were often aware of their transferable skills and could state them in the interview. They knew why they were applying for the position; how their background related; and the skills employers were probably looking for. For youth with disabilities, they often struggled with relating their skills and experience to the current position.

Based on our findings about providing feedback to group members and problem-solving scenario questions, youth with disabilities seemed to have fewer experiences in team-based environments. Youth with disabilities should be encouraged to participate in more group-based activities at school or through extra curricular activities to build up these skills. Clinicians and educators should help youth to find such accessible activities (e.g., volunteering, accessible sports, support groups etc.) that they can take part in.

The United Nations [24] suggests that attitudes about disability may contribute to reduced participation of youth with disabilities at school, work and local activities. Given the nature of their disability, youth may also be attracted to individual-based pursuits, such as doing well in school instead of participating in group-based activities, such as sports. Participation in sports or other extra curricular activities can help individuals with disabilities increase their self-esteem and opportunity for socialization [25]. Interestingly, research shows that people with disabilities who were physically active were more likely to be employed and reported higher life satisfaction [25]. Given that the most common industries employing youth are likely to be in the service sector [26], it is important that youth with disabilities develop the necessary interpersonal skills to succeed in team-based environments.

By using open-ended job interview questions, employers are trying to reduce their risk of making poor hiring decisions [13, 22–23]. Research shows that poor hiring decisions can have multiple negative effects on an organization, such as wasting financial resources, reducing team morale, and damaging company reputation [27]. When potential applicants refer to personal flaws, they are communicating to interviewers that they are unsuitable for the job. Applicants are instead recommended to speak about weaknesses that do not relate to the current position [13, 22–23, 27]. For example, speaking about poor communication skills is less important when applying for a position in computer programming than one in customer service. Interestingly, both groups of youth in our study provided potentially damaging responses to this question when they mentioned needing to improve their punctuality. This response might provide a negative impression to employers, such that these individuals will be unreliable and unproductive. Managing impressions and misperceptions is critical because the decision to hire an applicant is often made during the first four minutes of an interview [28].

### Implications for educators and health care practitioners

Our research has several implications for employment readiness programs and specifically interviewing skills for youth. First, youth with disabilities need to be coached on what transferable skills are provided by their experiences at school and work because employers expect workers to have good interview skills, which includes being able to market their skills and experiences [4, 19]. Second, youth with disabilities could benefit from more training on how to respond to open-ended questions, such as “What is your greatest strength?” Youth need to understand that employers are particularly interested in skills that relate to the current position. Third, employers need to be aware that the experiences of youth with disabilities may be different from that of youth with typical development. For example, youth with disabilities may have limited work experience, but they might possess valuable skills obtained through schooling or volunteer experience. Thus, employers should consider basing their impression of youth with disabilities on their collection of experiences instead of looking for a specific skill set. Fourth, all youth, should be given feedback about how they performed in an interview so they can work towards improving their job interviewing skills and future employment prospects [19]. Finally, clinicians should educate youth with disabilities on how and when to best disclose a disability [26].

### Strengths, limitations and future directions

A key strength of our research is that it provides a comparison of employment interviews among youth with disabilities and typical development. Most studies focus on adults with disabilities while our study explored youth and compared them to their typically developing peers. Our findings are important for educating employers about the challenges associated with interviews and informing employment readiness programs for youth with disabilities. Our study is limited in that we focused on individuals with physical disabilities, where in most cases the condition may be visible to employers. Future research is needed to understand the interview experiences of those with invisible disabilities, such as autism spectrum disorder and acquired brain injury. Second, we focused on verbal responses during an interview instead of non-verbal behaviour (e.g., facial expressions, body language), which can affect the meaning of a message and impressions formed during interviews [13]. Further research is needed to determine how verbal and non-verbal aspects of communication interact and affect an employer’s decision to hire. A third possible limitation is that we applied a content analysis, which tends to provide a less rich but more detailed analysis of some aspects of the data [18]. Although multiple data sources and methods were used in our larger study, our study here did not triangulate the data but rather focused only on the content of job interviews. Future research could explore job interviews in further depth, especially in areas where youth with disabilities may have differed from their peers.

Finally, future research should consider alternatives to structured job interview questions to capture the differing abilities and experiences of potential employees. For example, some companies including the Danish not-for-profit organization, *Specialisterne* have removed interviews from their hiring process in favor of using a performance-based approach [29]. It is important for companies to determine what the critical aspects of a position are and how these aspects will be assessed in job candidates.

## Conclusions

There were several similarities and differences in the content of job interview answers between youth with disabilities and typically developing youth. Similarities included giving examples from school and emphasizing their “soft skills” (i.e., people and communication skills) and giving examples of relevant experience for the position. Both groups of youth gave similar examples for something they were proud of but youth with disabilities gave fewer examples. Differences between the two groups of youth included youth with disabilities (1) disclosing their condition; (2) giving fewer examples related to customer and teamwork skills; (3) experiencing greater challenges in providing feedback to team members and responding to scenario-based problem solving questions; and (4) giving fewer examples from past work, volunteer and extra curricular activities.

Our findings can help to educate employers about the challenges associated with structured job interviews. In particular, these challenges include open-ended questions where responses should be relevant to the current position as well as applied and scenario-based questions where rationale should be provided to show their problem-solving ability. Our findings also help to inform employment readiness programs by highlighting the areas where youth with disabilities may need extra help with compared to typically developing youth.
